# Solution-state nuclear magnetic resonance studies of the *Salmonella typhimurium* tryptophan synthase complex

**DOI:** 10.1016/j.bbrep.2026.102586

**Published:** 2026-04-09

**Authors:** Rebecca N. D'Amico, Somnath Mondal, Dennis S. Winston, David D. Boehr

**Affiliations:** Department of Chemistry, The Pennsylvania State University, University Park, PA, 16802, USA

**Keywords:** Tryptophan synthase, Allostery, Enzyme-enzyme communication, Protein conformational dynamics, Methyl NMR spectroscopy

## Abstract

Tryptophan synthase (TS) is an allosteric bi-enzyme complex that catalyzes the final steps of l-tryptophan biosynthesis through tightly coupled α- and β-subunit reactions. Understanding how conformational dynamics mediate its catalytic efficiency and inter-subunit communication remains a key interest. Here, we establish that *Salmonella typhimurium* tryptophan synthase (*St*TS) is amenable to solution-state nuclear magnetic resonance (NMR) studies despite its large size (∼143 kDa). Towards this end, we optimized expression and labeling protocols to prepare ^13^CH_3_-δ1-Ile–labeled *St*TS and obtained high-quality NMR spectra, which enabled subunit-specific resonance assignments. Addition of both α- and β-subunit ligands propagate structural and dynamic changes across the entire complex, underscoring the high degree of inter-subunit communication. Our findings validate *St*TS as a tractable model system for solution-state NMR, which can provide new molecular-level insights into the mechanisms of substrate channeling, conformational dynamics and allosteric regulation in TS.

## Highlights

• Tryptophan synthase (TS) is a model system for understanding allosteric regulation.
• TS is a αββα heterotetramer with total molecular weight of 143 kDa.
• Methyl-based NMR spectroscopy allowed for high quality solution-state spectra.
• Subunit-specific resonance assignments were made.
• α- and β-ligand titrations confirmed long-range allosteric interactions.

## 1 Introduction

**Fig. 1:**
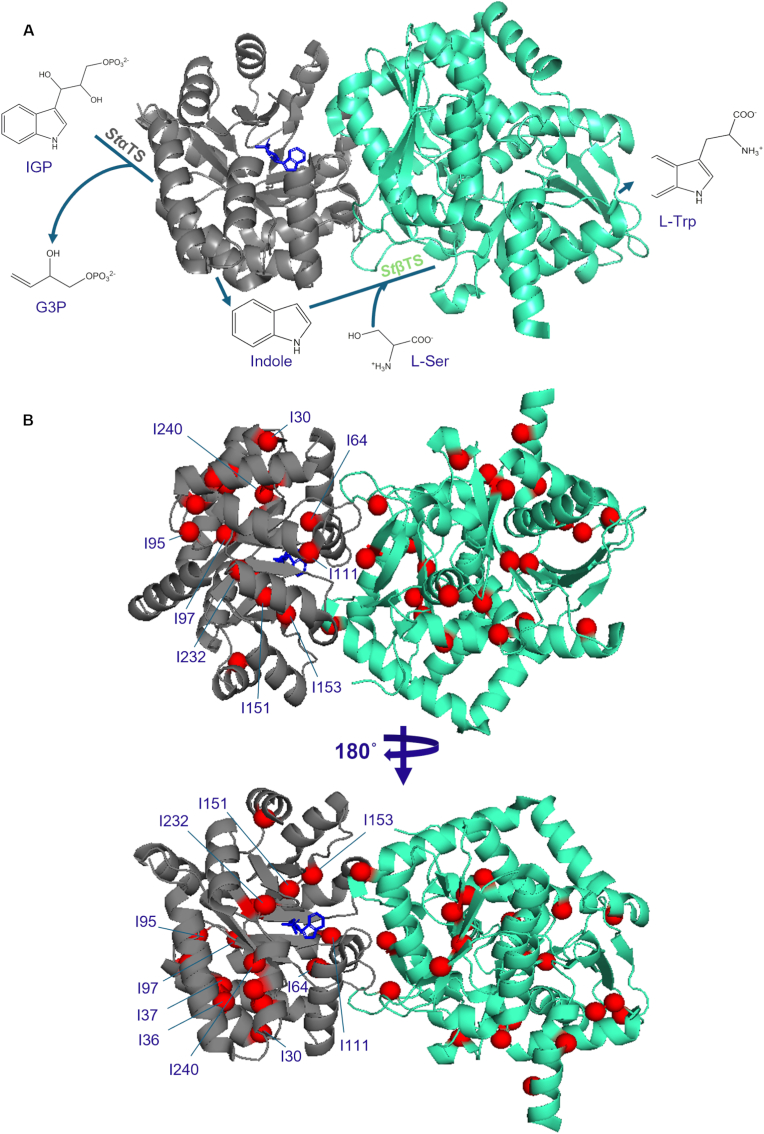
**Structure and catalytic function of tryptophan synthase.** (A) In the TS catalytic cycle, the alpha-subunit (αTS; grey) cleaves IGP using a retro-aldol reaction. The resulting indole is then channeled to the beta-subunit (βTS; green), where it combines with L-Ser to produce L-Trp. (B) Location of the Ile residues (red spheres) in *St*TS, which serve as NMR probes in this study. *St*TS is shown in two different orientations. PyMol was used in the creation of this figure using the *St*TS structure derived from PDB ID: 2RH9 with IGP shown in blue in the αTS subunit [12].

Tryptophan synthase (TS) is a bi-enzyme complex that catalyzes the final steps of tryptophan biosynthesis [[1], [2], [3], [4]], and serves as a classic model system for studying enzyme catalysis, substrate channeling, and allosteric regulation [[5], [6], [7], [8]]. The enzyme functions as a heterotetramer (α_2_β_2_), composed of two α-subunits (αTS) and two β-subunits (βTS) [[9], [10], [11]] (see Fig. 1). The α-subunit catalyzes the cleavage of indole-3-glycerol phosphate (IGP) to indole and glyceraldehyde-3-phosphate (G3P), while the β-subunit condenses indole with l-serine to form l-tryptophan [[9], [10], [11]]. Efficient substrate channeling between the two active sites prevents indole diffusion into bulk solvent and enhances catalytic efficiency [5,7]. TS is widely used to understand relationships between protein conformational dynamics, inter-enzyme communication and enzyme regulation [9,10,13].

TS and its individual subunits from both *E. coli* (*Ec*TS) and *S. typhimurium* (*St*TS) have been heavily studied (e.g. Refs. [3,6,14,15]). While early work focused on *Ec*TS, the first high-resolution X-ray crystal structure of the complete α_2_β_2_ complex was obtained for *St*TS [6]. Since then, *St*TS has served as a model system for probing the structural basis of allostery and conformational communication. It should be noted that *Ec*TS and *St*TS have high sequence similarity; *St*αTS shares 85% identity with *Ec*αTS, while *St*βTS is 97% identical to *Ec*βTS (Fig. S1–S2).

NMR spectroscopy has played a pivotal role in advancing our understanding of TS dynamics and allosteric regulation. Previous solution-state NMR studies have included those using *Ec*αTS as a model system to understand protein folding [16,17], and fluorine-based NMR studies on α-site ligands [18] to reveal insights into conformational equilibria, active-site chemistry, and communication between α- and β-subunits of *St*TS. The earlier efforts with *Ec*αTS laid the groundwork for understanding how protein motions may help tune catalytic efficiency and regulation. For example, our laboratory identified allosteric networks in *Ec*αTS proposed to be important for enzymatic function, although these studies were limited by the absence of the β-subunit [1,19,20]. Given the strong inter-subunit allosteric regulation in TS, extending NMR studies to the complete α_2_β_2_ complex was deemed essential for capturing functionally relevant structural and dynamic features. The combination of high sequence similarity to *Ec*TS, enhanced stability, and amenability to high-yield purification suggested that *St*TS might be suited for solution-state NMR characterization [21,22]. Indeed, *St*TS has been extensively studied by solid-state NMR [23]. Advances in fast magic-angle spinning and proton-detected methods enabled assignment of approximately 70% of backbone resonances for *St*TS in the solid state, with observations that such assignments can often be transferred to solution-state studies [21,24].

Methyl-based NMR spectroscopy provides a powerful approach for probing the structure and dynamics of large protein systems [[25], [26], [27]]. Favorable relaxation properties of methyl groups, including those of Ile, Leu, Val, Met, and Ala residues, extend the accessible size range of solution-state NMR into the megadalton regime [25,27]. Here, we collect and analyze NMR data of *St*TS in the solution state by focusing on Ile methyl probes, demonstrating that *St*TS is amenable to advanced solution-state NMR experiments. These results establish *St*TS as a model system for investigating allosteric regulation in tryptophan synthase and provide a platform for future studies of enzyme dynamics using solution-state NMR.

## 2 Materials and methods

### 2.1 Expression and purification of isotopically-labeled StTS complex

The heterotetrameric *St*TS complex was expressed using *E. coli* CB149 cells lacking endogenous tryptophan synthase using an adapted protocol from Hilario and coworkers [28]. A single colony was inoculated into 50 mL of NZCYM media (10 g/L casein peptone, 1 g/L casamino acids, 5 g/L yeast extract, 5 g/L NaCl, and 1 g/L MgSO_4_) and allowed to grow at 37 °C with shaking for 10 h. This culture (1 mL) was then used to inoculate 50 mL of D_2_O-based M9 media (14.196 g/L Na_2_HPO_4_, 6 g/L KH_2_PO_4_, 0.5 g/L NaCl, 3.0 g/L NH_4_Cl, 3.0 g/L deuterated glucose (C_6_D_12_O_6_), 4 mM MgSO_4_, 0.1 mM CaCl_2_, 1X MEM Vitamin Mix, 100 mg/L ampicillin, 15 mg/ml tryptophan all dissolved in D_2_O) [29]. The 50 mL culture was then allowed to grow at 37 °C for 16 h with shaking, before inoculating a 1 L of D_2_O-based M9 media. Large (1 L) cultures were grown at 37 °C with shaking until OD_600_ reached 0.5-0.6, following which 70 mg/L of labeled α-ketobutyric acid (Cambridge Isotope Laboratories, Inc. CLM-6164) was added. The cells were allowed to grow for 1 h to allow for incorporation of the isotope and were then induced with 0.25 g/L of IPTG (isopropyl β-*d*-1-thiogalactopyranoside**)**. Cell growth proceeded for 16 h at 30 °C with shaking.

Cells were harvested by centrifugation employing 4000×*g* for 30 min at 4 °C, and lysed using a digital sonifier using lysis buffer (50 mM Tris-Cl, pH 7.80, 100 mM NaCl, 5 mM dithiothreitol (DTT), 1 mM EDTA (ethylenediaminetetraacetate), and 1 mM PMSF (phenylmethylsulfonyl fluoride**)**). The cell lysate was then spun at 30,000×*g* for 30 min at 25 °C. The supernatant was aspirated and passed through a 0.45 μm filter at room temperature. The clarified supernatant was then subjected to a 20% ammonium sulfate fractionation at 25 °C, followed by another centrifugation step at 30,000×*g* for 10 min at 25 °C. This process was repeated for a 30% and 40% ammonium sulfate fractionation. The 40% ammonium sulfate fractionation pellet was resuspended in 10 mL of sample buffer (50 mM Tris-Cl, pH 7.80, 100 mM NaCl, 1 mM EDTA, and 2 mM DTT), and all fractions were analyzed using SDS-PAGE gel. The resulting fractions containing *St*TS were concentrated using a Corning Spin-X UF centrifugal concentrator with a 100 kDa cutoff (Sigma Aldrich, St. Louis, MO, USA).

### 2.2 Expression and purification of isotopically-labeled StαTS and StβTS subunits

The *St*αTS and *St*βTS subunits were separately expressed and purified based on an adapted protocol from Hilario and coworkers [28]. His-SUMO-tagged *St*αTS and *St*βTS were purified following the same protocol. Proteins were expressed using *E. coli* Rosetta (DE3) *pLysS* cells. A single colony was selected and grown for 16 h at 37 °C in 50 mL of NZCYM media. A portion (20 mL) of each culture was then inoculated into 1 L of NZCYM media and grown at 37^o^C shaking until OD_600_ reached 0.8. Cells were then collected through centrifugation (4000×*g* for 1 h). The resulting pellets were then re-suspended in H_2_O-based M9 media (14.196 g/L Na_2_HPO_4_, 6 g/L KH_2_PO_4_, 0.5 g/L NaCl, 3.0 g/L NH_4_Cl, 3.0 g/L deuterated glucose, 4 mM MgSO_4_, 0.1 mM CaCl_2_, 1X MEM Vitamin Mix, 100 mg/L ampicillin, 15 mg/ml tryptophan, all dissolved in H_2_O) and the supernatant discarded. Re-suspended cells were centrifuged again for 1 h at 4000×*g*. Resulting pellets were then resuspended in D_2_O-based M9 media containing 3 g/L deuterated glucose (C_6_D_12_O_6_). Cultures were then placed back in the shaker at 37 °C for 1 h before α-ketobutyric acid (140 mg/L, Cambridge Isotope Laboratories, Inc. CLM-6164) was added, and shaken for an additional hour at 37 °C. Protein expression was then induced with 0.25 g/L of IPTG, and expression occurred for 16 h at 30 °C [30,31].

Cells were collected using centrifugation (4000×*g* for 1 h) and lysed using a digital sonifier in lysis buffer (50 mM Tris-Cl, pH 8.0, 500 mM NaCl, 5% glycerol, 10 mM 2-mercaptoethanol (BME), and 40 mM imidazole-Cl). Lysed cells were spun at 30,000×*g* for 30 min and filtered through a 0.45 μM filter. Clarified supernatant was then applied to a 5 mL Ni-NTA column equilibrated in lysis buffer. *St*αTS and *St*βTS were eluted in a one-step process using elution buffer (25 mM Tris-Cl, pH 7.8, 200 mM NaCl, 5% glycerol, and 400 mM imidazole-HCl). The elution fraction was then subjected to a 60% ammonium sulfate fractionation to remove imidazole. The supernatant fraction was discarded, and the resulting pellet was resuspended in 20 mL of sample buffer (20 mM Tris-HCl pH 8.0, 300 mM NaCl, and 5% glycerol). The His-SUMO-tag was removed with Ubl-specific protease 1 from *S. cerevisiae* in a 1:1000 ratio. The cleavage was incubated for 16 h at 4 °C. The resulting cleavage product was spun at 10,000×*g* for 20 min at 25 °C to remove any protein aggregates. The resulting supernatant was passed through a second Ni-NTA column equilibrated in lysis buffer to remove the His-SUMO tag. The samples were concentrated using a Corning Spin-X UF centrifugal concentrator with a 30 kDa cutoff (Sigma Aldrich, St. Louis, MO, USA) to ∼1 mL and then applied to a Sephacryl S100 column (Cytiva) equilibrated in size exclusion chromatography (SEC) buffer (10 mM Tris-HCl, pH 7.8, 100 mM NaCl, 5% glycerol, and 0.1 mM PLP (pyridoxal 5′-phosphate)). Resulting fractions were characterized using SDS-PAGE and concentrated as described before.

### 2.3 NMR sample preparation and data collection

Purified proteins, including *St*TS, StαTS and StβTS were buffer exchanged into D_2_O-based NMR buffer (0.05 mM bicine, pH 7.80, 1 mM EDTA, 0.02 mM PLP, and 10 mM BME in D_2_O) using ZEBA desalting columns (Thermo Fischer). The StTS complex concentration was 850 μM, and StαTS and StβTS ranged from 150 to 300 μM, with 10 mM indole (Thermo Fischer), 10 mM G3P, 10 mM tryptophan, or 66 mM serine, where appropriate. The ligand concentrations were at least 5-10 times reported K_M_ or K_d_ values.

The *St*TS complex, *St*αTS, and *St*βTS were analyzed using SOFAST-HMQC [32] experiments performed on a Bruker Avance NEO 600 at 298K equipped with a 5 mm TCI single-axis gradient cryoprobe, with 32 scans and 96 indirect increments. The sweep widths were as follows: 1H, 5.0188 ppm; 13C, 15.0000 ppm. The methyl protons were excited with a center frequency of 1 ppm at a bandwidth of 3 ppm, using a flip angle of 120°.

## 3 Results and discussion

### 3.1 Comparison of the NMR spectra between the StTS complex and the isolated StαTS and StβTS subunits

**Fig. 2:**
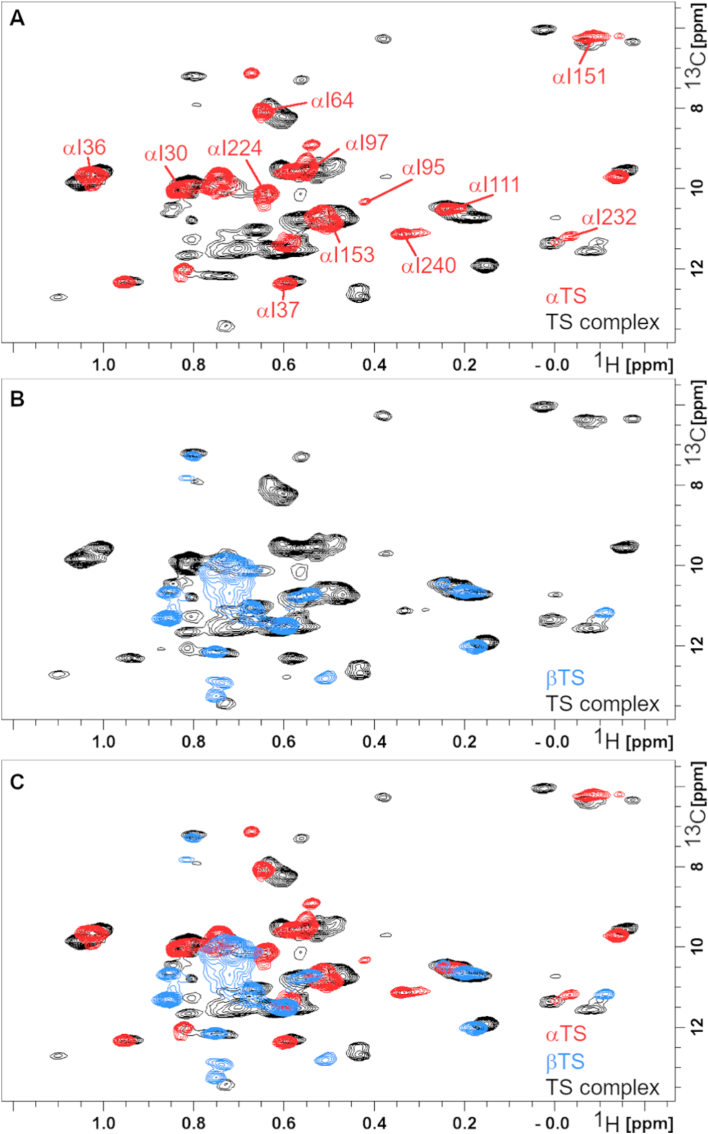
**δ1-^13^CH_3_ Ile NMR spectra reveal subunit-specific resonances and conformational dynamics in tryptophan synthase**. Comparison of spectra from the (A) monomeric *S*tαTS (red), (B) dimeric *St*βTS (blue) and the (C) full heterotetrametric *St*TS complex (black).

We collected SOFAST-HMQC NMR spectra for highly deuterated δ1-^13^CH_3_-labeled *St*αTS (monomeric, ∼30 kDa), *St*βTS (dimeric with total molecular mass of ∼83 kDa), and the full heterotetrameric *St*TS complex (total molecular mass of ∼143 kDa) (Fig. 2). The *St*αTS subunit contains 18 Ile residues (Fig. 1). However, we observe ∼23 δ1-^13^CH_3_ resonances in the StαTS spectrum, in which a few lower intensity resonances are near higher intensity resonances (e.g. resonance labeled as αI151 in Fig. 2A). This may suggest some heterogeneity in the protein sample (e.g. unprocessed His-tagged *St*αTS, partially liganded protein), although SDS PAGE did not indicate any substantial contamination of His-tagged or other proteins (Fig. S3), or perhaps sampling of a minor conformation. Further experiments, including more complete resonance assignments, may help to resolve this discrepancy.

For the *St*βTS subunit, we observe only ∼17 δ1-^13^CH_3_ resonances for the *St*βTS subunit, even though the β-subunit has a total of 24 Ile residues (i.e. missing ∼7 expected resonances; see Fig. 1). These residues, and others in *St*βTS, may experience motions on the intermediate NMR timescale, which acts to broaden their corresponding resonances beyond detection. Complex formation with the α-subunit may abrogate such motions to reveal the corresponding resonances in the *St*TS complex. Indeed, three Ile residues in the β-subunit are found near the α/β binding interface (Fig. S4).

For the *St*TS complex, we observe ∼46 δ1-^13^CH_3_ resonances, although the complex would be expected to have 42 unique Ile residues between the subunits. However, a few of these resonances are lower in intensity, which like the *St*αTS subunit, may suggest some sample heterogeneity (e.g. partially liganded protein), although SDS PAGE indicated a fully purified *St*TS complex (Fig. S3). Nonetheless, comparison of the *St*αTS, *St*βTS and *St*TS spectra allowed us to assign many of the *St*TS complex resonances to either the α- or β-subunits (see Fig. 2).

### 3.2 Combination of StαTS and StβTS does not entirely recapitulate the StTS NMR spectrum

**Fig. 3:**
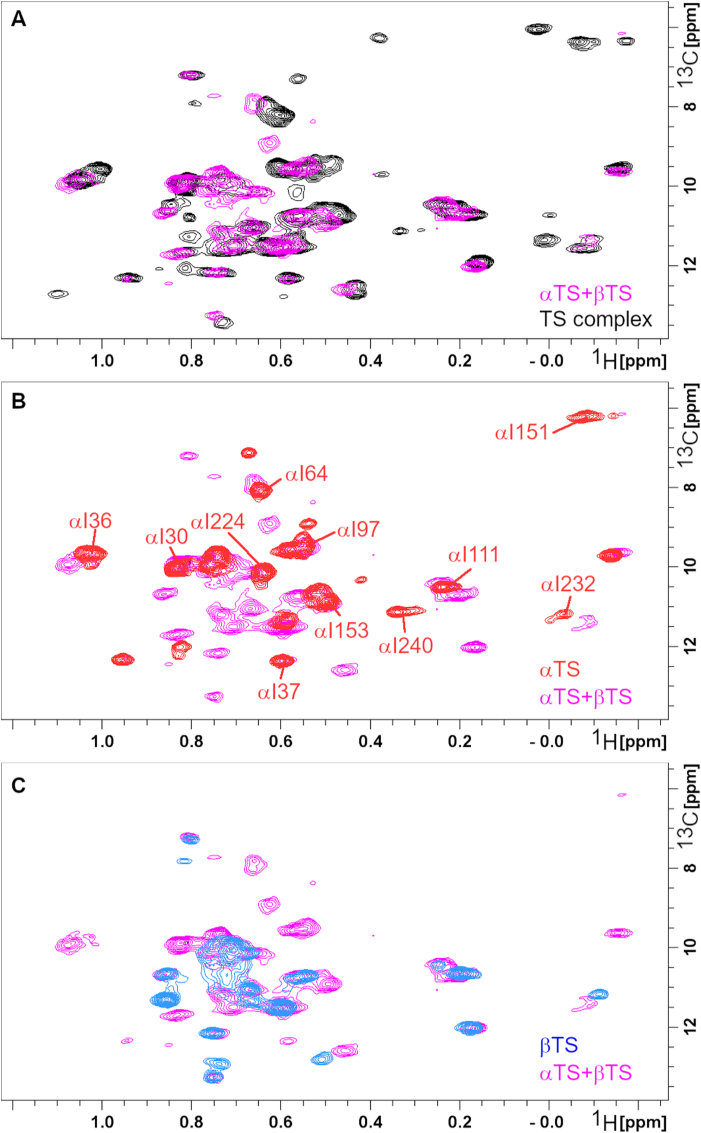
**Co-expression of the tryptophan synthase subunits leads to differences in NMR spectra compared to separately expressed, reconstituted tryptophan synthase.** Comparison of the ^1^H–^13^C SOFAST-HMQC spectra for (A) *St*TS in which subunits are co-expressed and purified (black) and separated expressed and reconstituted *St*TS (*St*αTS + *St*βTS; pink), (B) *St*αTS by itself (red) and separately expressed and reconstituted *St*TS, and (C) StβTS by itself (blue) and separately expressed and reconstituted *St*TS.

As future work may benefit from different labeling schemes for the two subunits (e.g. only one subunit isotopically labeled), we performed a preliminary investigation on the reconstituted heterotetramer, in which we combined the separately expressed/purified α- and β-subunits in a 2:1 α-monomer to β_2_-dimer ratio (Fig. 3). Addition of *St*αTS and *St*βTS subunits together led to some chemical shift changes, suggesting some conformational rearrangements upon complex formation. However, the resulting NMR spectrum still had missing resonances and other chemical shift differences compared to the co-expressed *St*TS complex (Fig. 3A). These results suggest that co-expression of the α- and β-subunits may give rise to structural and/or dynamic differences relative to complexes assembled by mixing the individually expressed and purified subunits. Alternatively, variations in the purification strategies used for *St*TS versus the isolated subunits could contribute to the observed differences, including the use of N-terminal His-SUMO tags for the separately expressed, but not co-expressed, subunits. Although the N-terminal His–SUMO tags are removed during purification of the individual subunits to restore their native N-termini, their presence during expression may still induce subtle effects on protein folding and/or structure. However, it is noted that the N-termini of both the α- and β-subunits are solvent-exposed and distal from the active sites and the α–β interface. A more thorough analysis of the functional, structural, and dynamic differences between the co-expressed and reconstituted heterotetramers may be necessary to clarify these effects, including disruption and reassembly of the co-expressed complex to directly assess contributions arising from differences in protein expression and purification.

### 3.3 Resonance assignments for StαTS

We have previously assigned most of the δ1-Ile resonances for *Ec*αTS [1,19]. Given the very high sequence similarity between *Ec*αTS and *St*αTS (Fig. S1), we proposed that most of these assignments would transfer to *St*αTS. In fact, the NMR spectra of *Ec*αTS and *St*αTS overlap very well (Fig. S5), allowing us to make tentative assignments for most of the *St*αTS resonances (Figs. 2a and 3b). Confirmation of these assignments and extension to the *St*βTS subunit (e.g. through ^13^CH_3_–^13^CH_3_ NOESY type strategies) will be necessary for a more thorough analysis.

### 3.4 Structural and/or dynamic changes in response to interaction with ligands

**Fig. 4:**
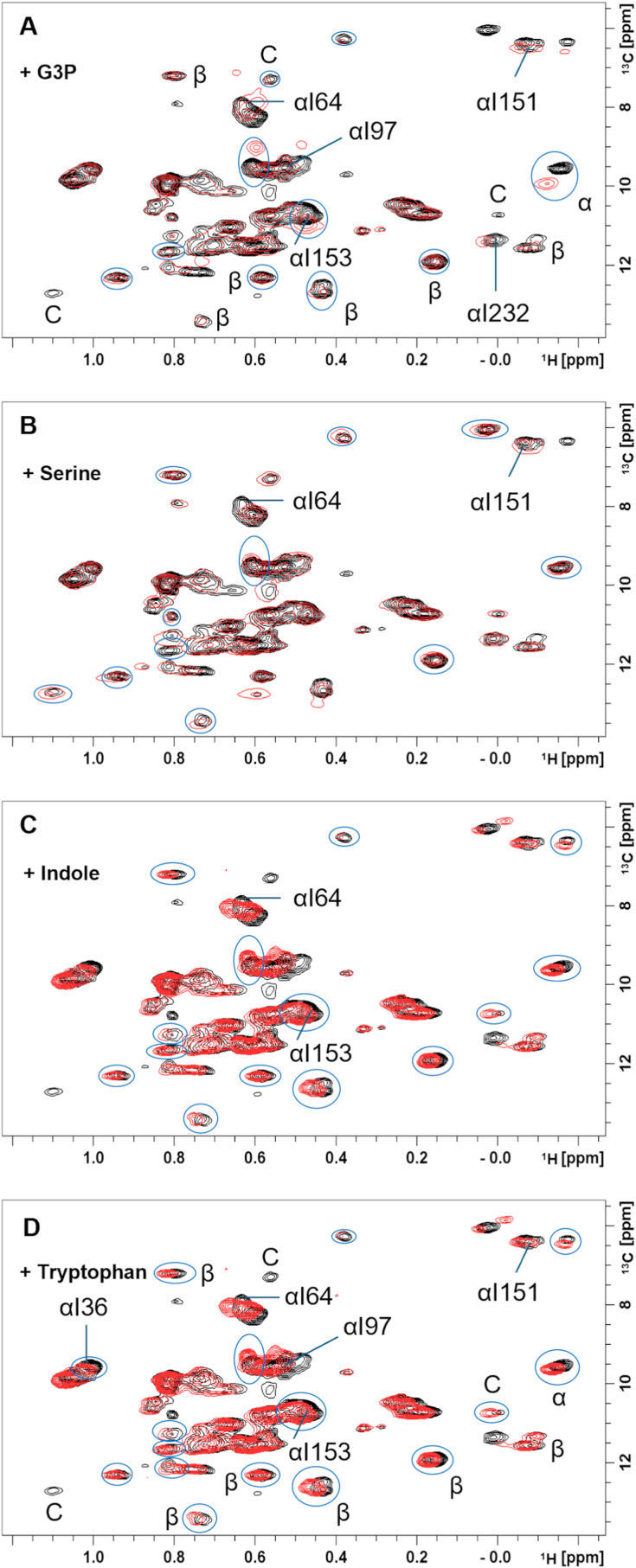
**Ligand-induced chemical shift perturbations in *St*TS reveal subunit-specific and complex-specific dynamics**. ^1^H–^13^C SOFAST-HMQC spectra of the *St*TS heterotetramer in the absence (black) and presence (red) of (A) glyceraldehyde-3-phosphate, (B) serine, (C) indole, and (D) tryptophan. The peaks that have undergone peak intensity and/or chemical shift changes are labeled and/or circled in the spectrum. α represents resonances assigned to *St*αTS, β represents resonances assigned to *St*βTS, and C represents resonances assigned to only the *St*TS complex.

To better understand solution-state structural and/or dynamic changes in *St*TS upon binding α- and β-subunit ligands, we collected NMR data (Fig. 4) for *St*TS in the presence of G3P (*St*αTS product), serine (*St*βTS substrate), indole (*St*αTS product, *St*βTS substrate), and tryptophan (*St*βTS product), which capture key states in the TS catalytic cycle. The addition of G3P induced chemical shift changes, including Ile64, Ile97, Ile151, and Ile232 in the α-subunit. Ile64, Ile151, and Ile232 face inward toward the StαTS active site (Fig. 4A), with Ile64 located in the hydrophobic binding pocket and likely experiencing structural changes upon G3P binding [33]. Ile97 lies on the β3 strand near catalytic residue Glu49. Previous work for *Ec*αTS also highlighted changes to the conformational dynamics of Ile151 and Ile232 upon G3P addition [5], which may also occur in the *St*TS complex. G3P also induced peak intensity and/or chemical shift changes in resonances associated with StβTS or unique to the StTS complex, suggesting structural or dynamic changes in the β-subunits, potentially near the α/β interface (Fig. 4A).

The presence of serine caused minor chemical shift changes, most associated with the β-subunit (Fig. 4B), consistent with its role as a *St*βTS ligand. However, two *St*αTS residues, αIle64 and αIle151, also experienced minor changes. It was shown previously that αIle64 resides in the hydrophobic binding pocket of *St*αTS, which undergoes conformational changes upon formation of the aminoacrylate in *St*βTS [34].

Indole is generated in the *St*αTS reaction and channeled to *St*βTS, where it is condensed with serine to form tryptophan. When indole or indole analogues are introduced to *St*TS, they appear to access the channel through the *St*αTS active site [35]. The presence of indole causes changes to resonances associated with both *St*αTS and *St*βTS subunits, as well as resonances unique to the *St*TS complex (Fig. 4C). These chemical shift changes are small, likely due to low binding affinity in the absence of serine. The resonance for αIle64 also shifts upon indole binding, likely reflecting its proximity to the *St*αTS indole-binding pocket.

When tryptophan is introduced in the *St*TS complex, chemical shift changes are observed for resonances associated with both *St*αTS and *St*βTS, as well as resonances unique to the *St*TS complex (Fig. 4D). Additionally, two resonances unique to the *St*TS complex disappear upon tryptophan binding. Tryptophan binding may induce conformational exchange, leading to resonance broadening; these resonances may correspond to Ile residues near the α–β binding surface. Tryptophan binding likely propagates structural changes across the *St*TS complex, altering the chemical environment of multiple Ile residues.

## 4 Conclusions

Previous solution-state NMR studies of the αTS subunit have provided insight into allosteric networks and conformational dynamics but were limited by the absence of the βTS subunit. Building on prior solid-state NMR work, we extend these efforts to solution-state NMR using methyl probes, which are well suited for large protein systems. We acquired ^1^H–^13^C SOFAST-HMQC spectra of isolated *St*αTS monomer, *St*βTS dimer, and the full heterotetrameric complex, enabling subunit-specific assignments and interpretation of spectral differences in terms of dynamics at the α/β interface. While all Ile resonances were detected in *St*αTS, several were absent in *St*βTS, consistent with intermediate time-scale exchange; complex formation restored many of these signals, suggesting conformational stabilization upon assembly at the α/β interface.

## Funding

This study was supported by NSF grant MCB 1615032.

## CRediT authorship contribution statement

**Rebecca N. D'Amico:** Conceptualization, Formal analysis, Investigation, Methodology, Writing – original draft. **Somnath Mondal:** Formal analysis, Visualization, Writing – original draft, Writing – review & editing. **Dennis S. Winston:** Investigation, Methodology, Writing – review & editing. **David D. Boehr:** Conceptualization, Funding acquisition, Project administration, Supervision, Writing – original draft, Writing – review & editing.

## Declaration of competing interest

The authors declare that they have no known competing financial interests or personal relationships that could have appeared to influence the work reported in this paper.

## Data Availability

Data will be made available on request.
