# Supplementary material for: Solution-state nuclear magnetic resonance studies of the Salmonella typhimurium tryptophan synthase complex
Source: Biochem Biophys Rep. 2026 Apr 9;46:102586. doi: 10.1016/j.bbrep.2026.102586 (PMC13091236; doi:10.1016/j.bbrep.2026.102586)
Supplement: Multimedia component 1 [file mmc1.docx]

***Supplementary Information for:***

**Solution-state nuclear magnetic resonance studies of the *Salmonella typhimurium* tryptophan synthase complex**


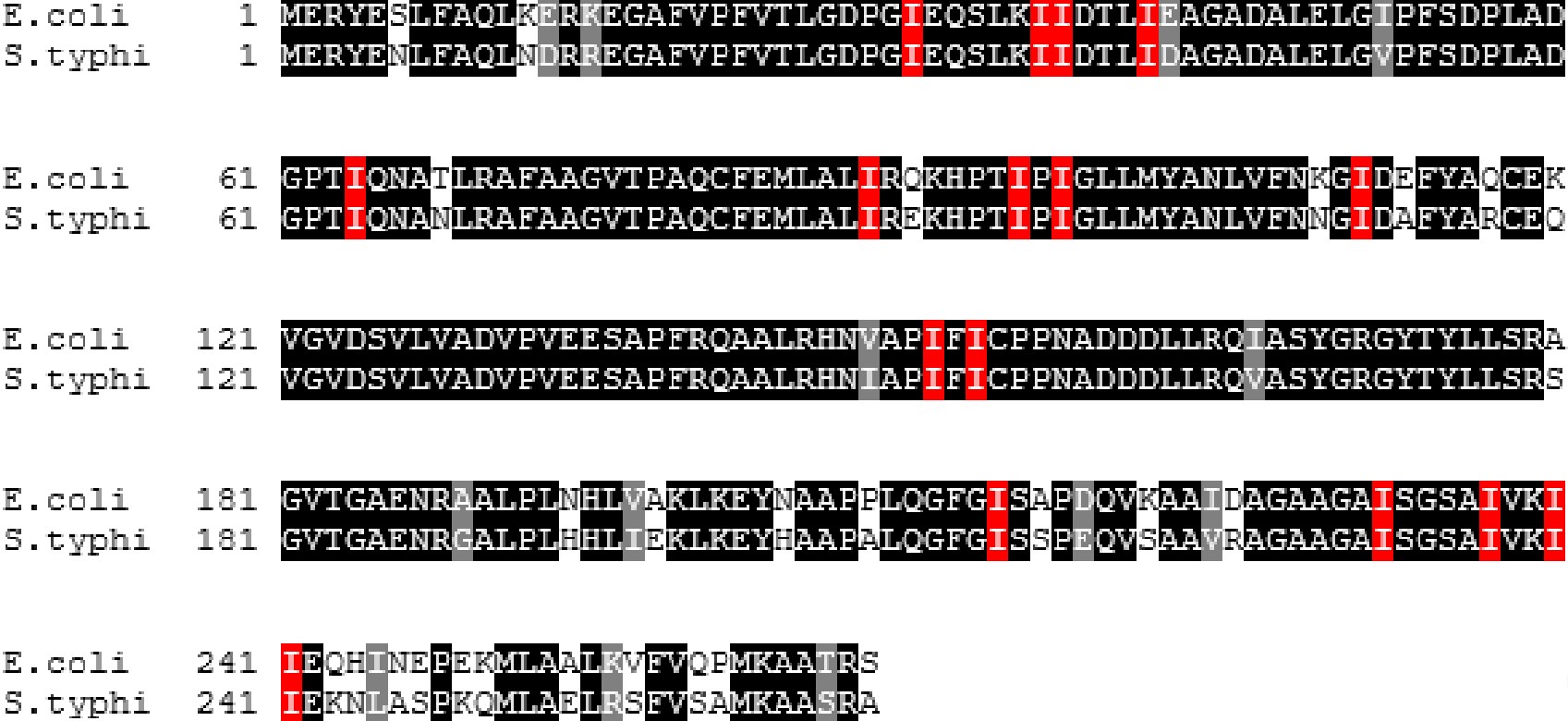


**Figure S1. Sequence alignment between EcαTS and StαTS.** Residues highlighted in black are identical in both sequences. Residues highlighted in grey indicate conservation between amino acids with similar properties. Conserved isoleucine residues (16) are highlighted in red. EcαTS and StαTS are 85% identical in sequence.


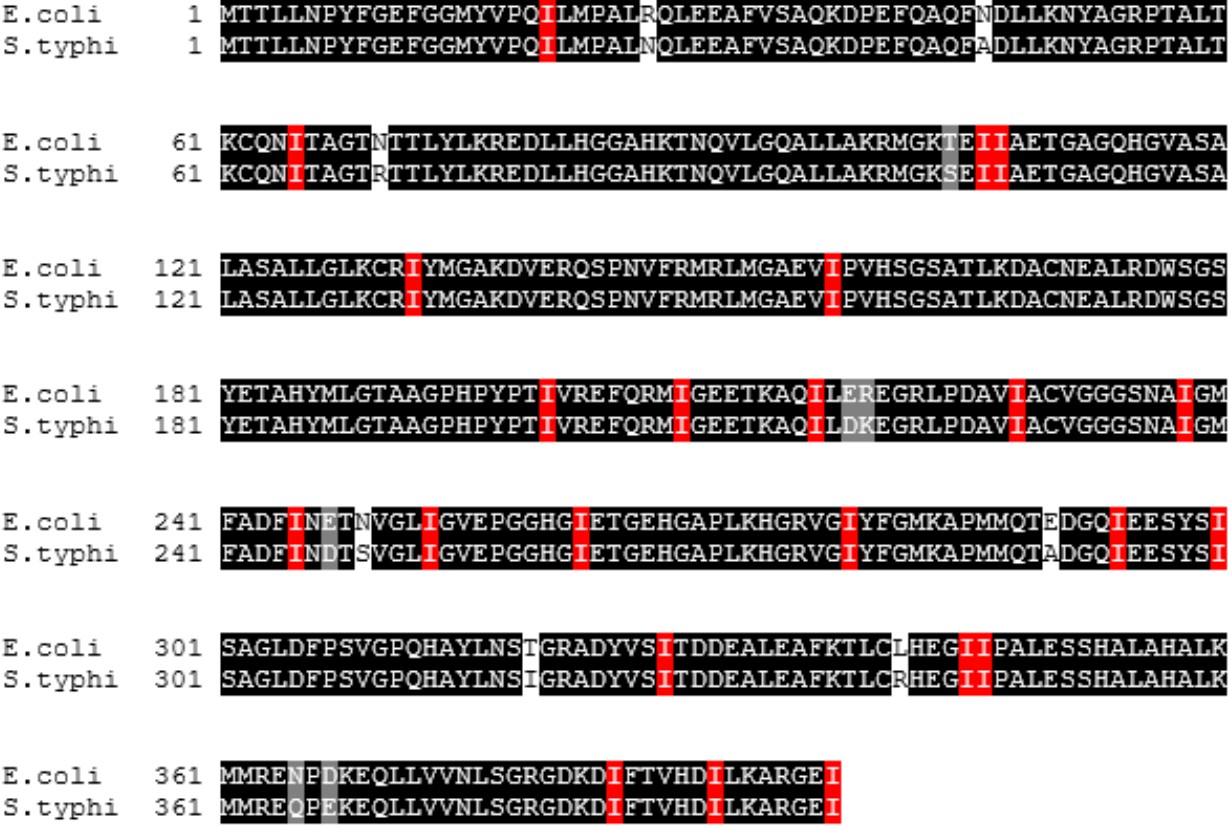


**Figure S2. Sequence alignment between EcβTS and StβTS.** Residues highlighted in black are identical in both sequences. Residues highlighted in grey indicate conservation between amino acids with similar properties. Conserved isoleucine residues (23) are highlighted in red. EcβTS and StβTS are 97% identical in sequence.


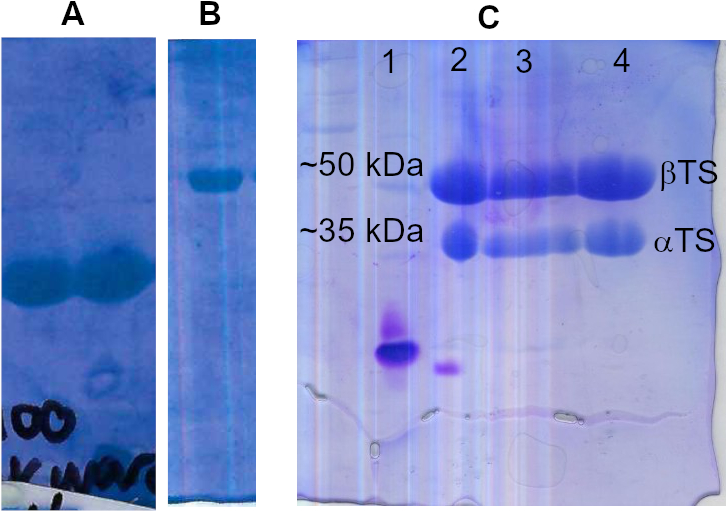


**Figure S3. Purity of the isolated *St*αTS and *St*βTS subunits and the co-expressed *St*TS complex.** (A) SDS PAGE showing purity of *St*αTS following Sephacryl S100 size exclusion chromatography; lanes represent different fractions that were pooled for further analysis. (B) SDS PAGE showing purity of *St*βTS following Sephacryl S100 size exclusion chromatography. (C) Purification of the *St*TS α_2_β_2_ heterotetramer showing samples corresponding to the supernatant (lane 1) and precipitant (lane 2) of the 40% ammonium sulfate cut, resuspended protein following ammonium sulfate precipitation (lane 3) and protein following NMR sample preparation (lane 4). Approximate sizes of molecular weight markers (not shown) are indicated. Gels A, B and C were run and processed under different conditions and cannot be directly compared.


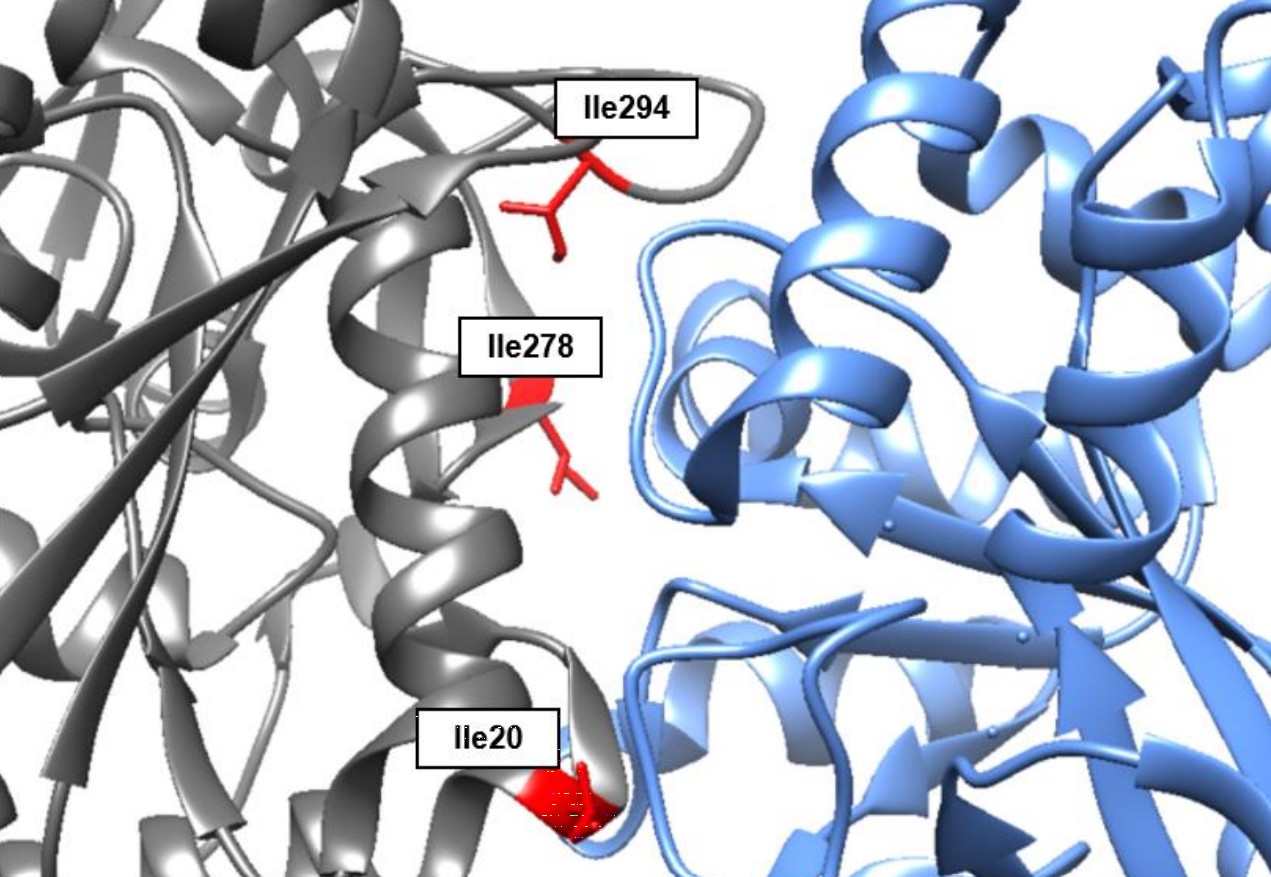


**Figure S4. The α/β binding interface in StTS.** StαTS is colored blue, StβTS is colored grey. Three StβTS Ile residues are found near the binding interface – βIle20, βIle278, and βIle294. This figure was based on PDB ID: 2CLK.


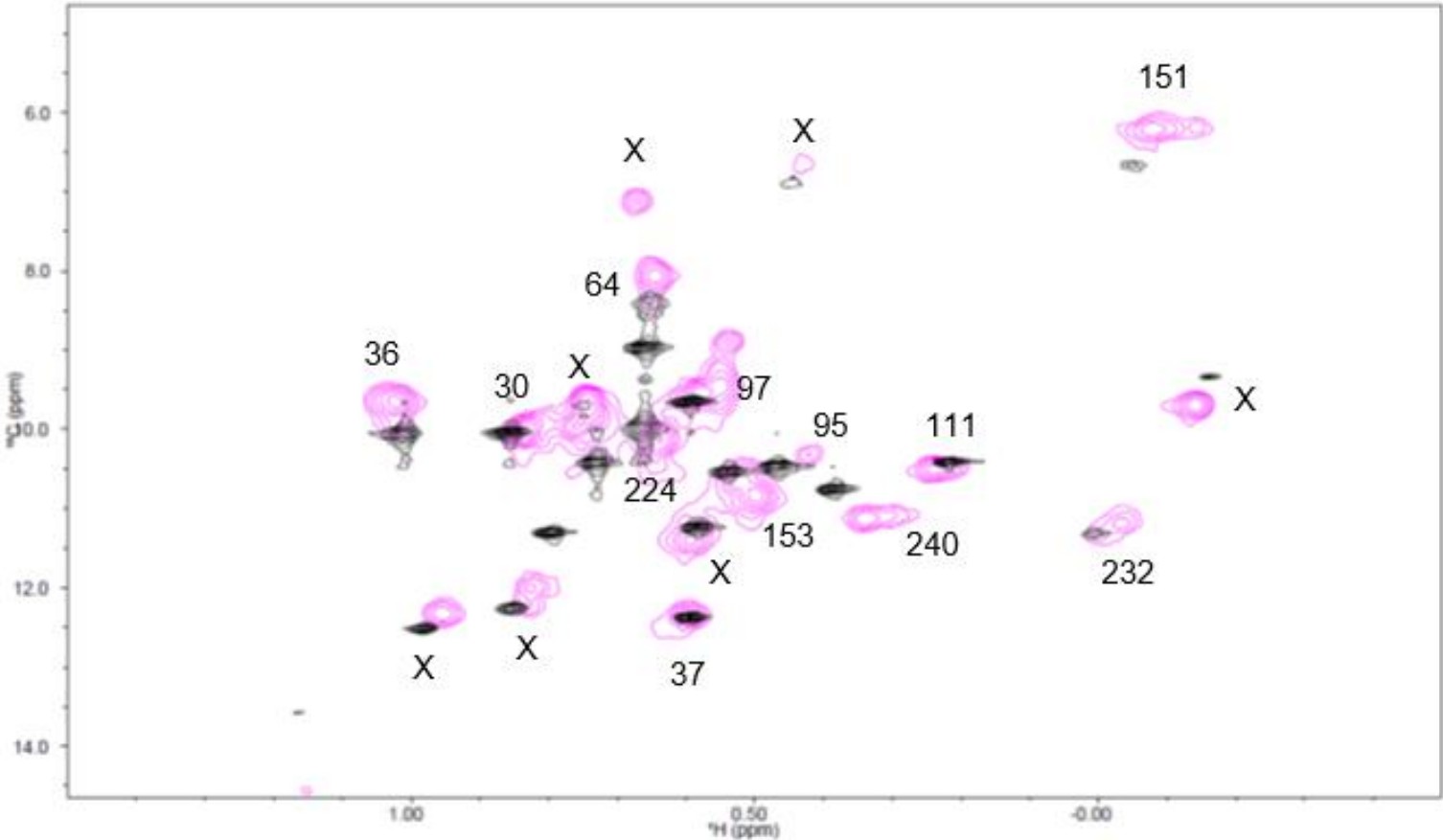


**Figure S5. Comparison of the ^1^H- ^13^C HMQC spectra for StαTS (pink) and EcαTS (black).** ^1^H- ^13^C HMQC spectra for StαTS (pink) and EcαTS (black) are compared. Only Ile residues are labelled. X resonances are unassigned.
